# Associations between socio-demographic patterns, body dissatisfaction, and eating disorder risk in women: a cluster-based approach

**DOI:** 10.1186/s40337-026-01616-8

**Published:** 2026-05-11

**Authors:** Vanessa C. Jürgensen, Georg Halbeisen, Martin S. Lehe, Georgios Paslakis

**Affiliations:** Ruhr-University Bochum, University Clinic for Psychosomatic Medicine and Psychotherapy, Medical Faculty, Campus East-Westphalia, Virchowstraße 65, 32312 Luebbecke, Germany

**Keywords:** Eating disorder risk, Socio-demographic patterns, Body dissatisfaction

## Abstract

**Objective:**

This study examined the association between socio-demographic patterns and eating disorder (ED) risk in 298 women (mean age = 28.4 years). We focused on women, as existing research suggests that EDs disproportionately affect women. Within this sample, we took into account the intersections of different socio-demographic variables. Additionally, we assessed body dissatisfaction and subjective health status (S-HS) as self-reported measures to gain a more comprehensive understanding of ED risk.

**Method:**

We conducted a cluster analysis (k-means) using ten demographic variables (e.g., sexual orientation, migration history, presence of disabilities), which revealed three distinct participant clusters. Then, we applied two multiple logistic regression models using cluster membership, body dissatisfaction related to fat (BD-F) and muscularity (BD-M), and S-HS as determinants, with the outcome being ED risk measured using two scales (EAT-8; EDE-Q).

**Results:**

Cluster Three – notably characterized by queer women with a migration history and identification as part of an ethnic minority - showed a consistently higher ED risk. In contrast, Cluster One, which included a higher proportion of older individuals as well as individuals with disabilities, or caregiving responsibilities, showed the lowest risk for ED. In Cluster Two an increased risk for EDs was observed in the EAT-8, but not in the EDE-Q, suggesting measurement-specific differences. BD-F and BD-M were significantly associated with ED risk. BD-F proved to be the factor with the strongest influence.

**Conclusions:**

We emphasize the importance of considering person-centered socio-demographic positions and different forms of body dissatisfaction to assess the risk of ED.

**Supplementary Information:**

The online version contains supplementary material available at 10.1186/s40337-026-01616-8.

## Abstract (plain language summary)

This study explored how different social and personal factors relate to the risk of developing an eating disorder (ED) in almost 300 women. We looked at how variables, such as sexual orientation, cultural background, migration history, and disability, are grouped together and might affect this risk. We found that one group, including more queer women, women with migration history, and those from ethnic or racial minority backgrounds, had a higher risk of EDs. In contrast, another group, mainly consisting of older women and more participants with disabilities and caregiving responsibilities, showed a lower risk. A third group showed a mixed pattern, highlighting possible differences between the measurement tools. Feeling unhappy with body fat or muscle tone also increased ED risk, especially dissatisfaction with body fat. Overall, our findings show that ED risk depends on both socio-demographic variables and body image. Taking into account structural differences linked to socio-demographic factors and body dissatisfaction may help improve prevention and support for women at higher risk.

**Supplementary Information:**

The online version contains supplementary material available at 10.1186/s40337-026-01616-8.

## Background

Eating disorders (EDs) are characterized by body image concerns, altered eating patterns, and weight-control behaviors [1]. EDs often develop in adolescence with a peak onset age around 15 years [2] and are highly comorbid with anxiety disorders (up to 62%), mood disorders (up to 54%), substance use (up to 27%), and post-traumatic stress disorders (up to 27%) [3]. Given the resulting burden and elevated mortality rates [3], identifying individuals at risk for EDs is crucial, as well as recognizing risk factors and early signs.

Men and women are differently affected by EDs, with women showing a substantially higher lifetime prevalence (8.4% vs. 2.2%) [4]. However, it should be noted that emerging evidence suggests EDs in men are often underrecognized and underdiagnosed due to stigma and gender biases in research and clinical practice [5]. Nevertheless, developing an ED has predominantly been associated with striving towards the ‘thin-ideal’ of (women´s) bodies that is prevalent in the Western-industrialized world. The term ‘thin-ideal’ gained traction as part of feminist and body image research in the 1990s, when scholars and activists began to examine the harmful effects of media representations on women’s body image [6]. This has partly contributed to the fact that ED research and interventions have tended to target a social group colloquially known as the ‘SWAG’, i.e., ‘skinny, white, affluent, girls’ [7]. However, this may overlook large groups of people also affected by EDs and underestimate the diversity of risk factors for EDs, including among women.

### Challenging the ‘SWAG’ stereotype

Recent research has increasingly focused on how populations may be at higher or lower risk for EDs due to social and structural inequalities. For example, there is growing evidence of racial and ethnic inequalities in the field [7, 8]. In the context of the US, a study found that female students from ethnic minority backgrounds, e.g. Black Hispanic/Latino Asian/Pacific Islander AI/AN/NH* Bi/Multiracial were less likely than White female students to use dieting or exercise for weight loss but more likely to engage in extreme weight-control measures, which underlines the need for culturally sensitive research [9]. European studies are less likely to report ethnicity than those conducted in the United States [10], reflecting the specific historical, political, and social context in many European countries, where collecting racial or ethnic data is often avoided [11]. At the same time, there is growing evidence that migration can be a risk factor for EDs as adapting to a new cultural environment may increase stress and intensify body image concerns [12]. The recording of variables relating to ethnicity or migration history is multidimensional. Therefore, one suggestion is to consider multiple variables, e.g., migration history and language skills as well as a more broadly applicable variable, asking about being part of a minority group [13]. Minority in this context refers to individuals who belong to groups that are numerically smaller or socially marginalized within a given society, which may influence their exposure to discrimination, access to resources, and overall health outcomes. Limited language proficiency may restrict access to healthcare and increase the likelihood of discrimination—ultimately contributing to poorer mental and physical health outcomes and migration-related-stress [14].

Reviews have also pointed to the increasing awareness of the vulnerability of the LGBTQ+ population to EDs [7, 15]. Emerging research increasingly suggests that (chronic) minority stress [16] places LGBTQ+ individuals at particularly high risk for EDs as well as body image concerns [17, 18]. Minority stress appears to influence EDs through several psychological pathways, including negative affect, social anxiety, social isolation, and the internalization of appearance ideals [18]. At the same time, there is evidence that aspects of LGBTQ+ community belonging can have a protective effect [17, 19].

One specific aspect of the persistent SWAG misconception, that EDs primarily affect wealthy girls, has shaped both research and public perception for decades [20, 21]. Consequently, experiences by individuals with low socio-economic status (SES) have often been overlooked. Against this backdrop, SES deserves particular attention. SES is an important determinant of health and can be conceptualized in both objective and subjective terms. Objective SES refers to measurable indicators such as education, income, or occupation, whereas subjective SES captures individuals’ perceived social and economic standing within society [22]. Research shows that EDs occur across all levels of objective SES [21]. Findings from Germany indicate that prevalence rates of suspected EDs among adults were not associated with educational attainment, but rather with lower subjective social status [23]. In general, lower perceived socio-economic standing has been linked to reduced well-being [24] and an increased risk of developing EDs [25]. Moreover, food insecurity, which is associated with low SES, has also been associated with a heightened vulnerability to EDs [20], suggesting that material and psychosocial stressors might be risk factors.

### Body dissatisfaction

A theory from the 1990s, the Tripartite Influence Model (TIM), states that EDs are influenced by three main factors: peers, parents, and the media [26]. These factors influence the development of body dissatisfaction and resulting EDs through the psychological mechanisms of internalizing beauty ideals and body comparison. The model has had a significant impact on research in the field of EDs, as it focuses on psychosocial influences and body dissatisfaction as key risk factors. EDs have traditionally been framed around the pursuit of thinness; however, this understanding has expanded to include concerns about muscularity, observed among men [27], but increasingly reported among women as well. Muscularity concerns reflect pressures to attain a body that is not only lean but also toned and strong, linking to both appearance-based and performance-based ideals. In general, body ideals are shaped by overlapping social and cultural identities [28]. For instance a study argued that the drive for thinness has historically been associated with whiteness, whereas the valorization of “thickness” as a marker of Black femininity and pride can be understood as a challenge to hegemonic standards of beauty [29]. Within queer and lesbian communities, identity terms such as “butch,” “femme,” “tribe,” and “dyke” carry specific meanings related to body expression, thereby shaping distinct body ideals, not only related to thinness, but also to muscularity [7]. These updated findings underscore the need to examine how not only the pursuit of thinness, but also muscularity concerns intersect with identity-specific pressures to influence ED risk. Similarly, a study found that queer women attribute body dissatisfaction to an interplay of personal, interpersonal, and social factors [30].

To sum up, beyond minority stress, migration-related stress, and socioeconomic hardship, body dissatisfactions also play an important role in relation to EDs. Studies show that risk factors for EDs include early trauma and stressful life events [31], but genetic, autoimmune, and personality-related influences as social pressure and competitive sports, also play a role [32]. This underscores the heterogeneity of etiological pathways [33] and emphasizes the importance of real-life circumstances, social position, and associated psychosocial vulnerabilities in the development of EDs.

### Towards consistent measurement of socio-demographic variables in ED studies

While much valuable focus has been placed on factors such as migrant populations, social status, and the LGBTQ+ community, other aspects of socio-demographic variables remain unexplored in ED research. In addition, there is also a lack of standardized methods for capturing multiple socio-demographic variables. For this reason, Stadler et al. developed the Diversity Minimal Item Set (DiMIS) as a tool for standardizing socio-demographics [13]. Accordingly, they suggest asking about informal care work. Informal care work interacts with gender, sexual orientation, and age [34] and has been associated with worse mental health [35]. Relative to formal carers, informal carers report worse nutritional outcomes, greater food insecurity, and higher stress, pointing to an important axis of social inequality [36]. Stadler et al. also suggest asking about the general subjective health status (S-HS) and the presence of disabilities. Physical disabilities have a significant impact on health determinants [37] and body image issues in the context of disabilities are associated with the development of EDs [38]. Studies on disabilities and eating disorders have examined, in particular, the concerns of those affected in relation to feeling different and the resulting psychological stress [39]. In a similar vein, it has been proposed that a multidimensional model for the relationship between disability status and EDs should be used for a routine assessment of the presence of disabilities [40].

### The present research

Consistent with recent recommendations [13, 25] this study adopted an approach that incorporated 10 key socio-demographic factors in addition to body dissatisfaction to explore ED risk in women. We specifically chose to examine the role of socio-demographic variables in women, as women continue to be the group most at risk for EDs [4]. We first explored how the ten factors interact to form distinct clusters. We chose a cluster-based, person-centered approach, which has multiple advantages. Firstly, it enables the collection of a broader set of socio-demographic data that more accurately reflects real-life circumstances rather than treating them as isolated predictors. Secondly, cluster analysis allows us to group the data, thus reducing data complexity while preserving data depth. Thirdly, cluster analysis helps identify specific risk groups, enabling us to focus on populations that warrant further attention in ED research. In a second step, alongside the person-centered socio-demographic clusters, we identified body dissatisfaction (both body fat- (BD-F) and muscularity-related (BD-M)), and further subjective variables, namely subjective socio-economic status (S-SES) and subjective health status (S-HS), for the prediction of ED risk. We aim to consider different perspectives in ED research by examining socio-demographic positions and body dissatisfaction in relation to ED risk.

## Materials and methods

### Participants and procedure

This is a planned secondary analysis of an online cross-sectional survey [41]. The original study recruited participants aged 18 years or older who identified as women, living in Germany. Details on the sample size and its rationale are provided in the original study [41]. The sample was recruited via Prolific (https://www.prolific.com/) and through advertisements on our website, university mailing lists, and personal networks between May 8th and June 27th, 2024. The original study targeted at least 300 participants and had been reviewed and approved on March 15th, 2024, by the ethics committee (reference number: AZ 2022-910_1). The present analysis had also been pre-registered before the original data collection on the Open Science Framework (OSF, https://osf.io/efn4v). The study was conducted in accordance with the ethical principles outlined in the Declaration of Helsinki, with all participants providing informed consent. Data were pseudonymized and linked to unique study IDs, ensuring anonymity; researchers had no access to personal identifiers, and only demographic or screening data were shared for eligibility purposes. The study was implemented in jsPsych [42]. After participants accessed the study website, they reviewed the study information and consent forms. Participants were informed that the focus of the study would be on exploring disordered eating behaviors in women, and they had the option to receive feedback on their individual ED risk. Participants recruited via Prolific were compensated £4.50 for their participation. The other participants did not receive any monetary compensation, but eligible university students received course credits. Since financial incentives can encourage the use of bots, recent research has outlined several strategies for reducing bot activity and low-quality responses in online surveys. These include, e.g., attention checks, the identification of speeders, the request of an email address, the request for photo-evidence of eligibility, and the use of questions requiring more attention (such as „theory of mind“ questions) [43]. As no single method is sufficient on its own, combining multiple strategies is recommended. Prolific already includes onboarding checks, such as phone and email verification, ID verification using a live video selfie, and VPN restrictions; in addition, researchers can reject exceptional fast speeders. We implemented our own safeguards by including an attention check and an open-end feedback question at the end of the study. We excluded participants who indicated male gender and who failed to pass the attention check, as well as individuals marked as „exceptionally fast“. Completion times ranged from 7.75 to 107 min (M = 23.09, SD = 11.80). Within the survey, participants were asked again to indicate their gender identity. This procedure ensured the inclusion of women as well as participants who indicated female gender, and additional self-identities beyond the gender binary of man and woman.

### Measures

#### Socio-demographic variables

We focused on ten socio-demographic aspects. First, participants were asked again to indicate their gender identity by responding to the question “Which gender do you feel you belong to?” with the options: *man*,* woman*,* diverse*, or *no answer*. We asked participants about their age (“How old are you?“), sexual orientation (“Who are you attracted to?“; *men*,* men and women*,* women*,* other genders*,* no answer*), migration history (*yes/no*), German language skills (*native*,* fluent*,* basic knowledge*), and identification as an ethnic minority or a racialized group (*yes/no/I don’t know*). We also inquired presence of disability (*yes/no/no answer)* and informal care work (e.g., *yes/no for caring for one or more adults or children*). Finally, participants were asked to report their years of schooling (*less than or at least 12 years*) and their family status (*single*,* married/in partnership*,* divorced).*

#### Subjective health status (S-HS) and subjective socio-economic status (S-SES)

To account for subjective factors, we asked participants about their subjective (general) health status (S-HS): “How would you rate your state of health in general?” with the following response options: *very good*,* good*,* fair*,* poor*,* very poor*,* and not specified*. We also asked participants a question about their subjective socio-economic status (S-SES) using the MacArthur Scale [44]: “Imagine a ladder with 10 rungs displaying where people in Germany stand. At the top rung (#10) are the people with the most money, the most education, and the best jobs. At the bottom rung are those with the least money, the lowest education, and the worst jobs or no job (#1). The higher you position yourself on the ladder, the closer you are to the people at the top; the lower on the ladder you think you are, the closer you are to the people at the bottom. Where would you place yourself on the ladder?” Responses were provided on a scale from 1 to 10. We also gave participants the option to skip the question if they preferred not to answer.

#### EAT-8

We used the Eating Attitudes Test–8 (EAT-8) to assess eating disorder (ED) risk [45]. The EAT-8 is a well-established screening instrument assessing concerns and preoccupations related to dieting, body image, and weight. It consists of eight items with dichotomous responses (1 = “I agree somewhat”, 0 = “I disagree somewhat”). In women, a score of ≥ 2 has been validated as indicating elevated risk for an ED [45]. The scale showed acceptable internal consistency in the present sample (Cronbach’s α = 0.79).

#### EDE-Q

Additionally, we administered the validated German version of the Eating Disorder Examination–Questionnaire (EDE-Q; [46, 47].” The EDE-Q assesses cognitive and behavioral eating disorder symptoms over the past 28 days. It consists of 22 attitudinal items rated on a seven-point scale (0 = “never” to 6 = “every day”), plus six additional questions addressing further eating disorder–related behaviors. As prior research has not supported the proposed factor structure [48], only the global mean score across the attitudinal items was used. Internal consistency in the present sample was excellent (Cronbach’s α = 0.96). We applied a global EDE-Q cut-off score of ≥ 2.4, representing the upper end of the empirically suggested range for discriminating between clinical and non-clinical at-risk cases [49], to complement the EAT-8 as a secondary screening indicator that is more stringent.

#### Body dissatisfaction

We used the Female Body Scale (FBS) and the Female Fit Body Scale (FFITBS) as figural rating scales designed to measure participants’ body dissatisfaction regarding muscularity (BD-M) and body fat (BD-F) [50]. We used body dissatisfaction as a surrogate for participants’ internalized body ideals. The FBS depicts a series of nine women´s bodies ranging from emaciated to obese, while the FFITBS shows a series of nine women’s bodies ranging from emaciated to very muscular. We asked the participants to indicate which body best represented their current body and to select their preferred bodies. For analyses, we used the absolute value of the discrepancy between the actual and ideal body for both BD-F and BD-M. Consequently, the scores in both scales could range from 0 (no body dissatisfaction) to 5 (greatest body dissatisfaction).

#### Additional questions

Participants provided additional information on their weight and height, medication use, menstruation status, history of EDs, living circumstances, and (ongoing) treatment for EDs. We further included an attention check question at a random position in the survey (“Please mark the word “giraffe” from a series of options) to ensure the integrity of the data. Other psychometric questionnaires included in the study are described elsewhere [41, 51].

#### Data analysis

We conducted all analyses using R version 4.4.2 (*R: The R Project for Statistical Computing*). The following packages were used for data manipulation, visualization, statistical modeling, and clustering: *dplyr*, *cluster*, *ggplot2*, *tidyr*, *sjPlot*, *car*, *factoextra*, and *gridExtra.* We computed descriptive statistics (*N* and *%)* for most demographic variables, such as gender, age, sexual orientation, migration history, identification as part of an ethnic minority or racialized group, education (years of schooling), family status, informal care work, and disability. For age, we calculated the *mean*,* Standard Deviation (SD)*, and *range.*

For the main analysis, as outlined in the pre-registration in the Open Science Framework, we conducted a cluster analysis to identify groups of participants with similar socio-demographic patterns. However, we opted for k-means clustering instead of Latent Class Analysis (LCA) as preregistered. LCA assumes that the observed variables can be explained by latent, probabilistic classes and relies on the assumption of conditional independence among the indicators. Because the sociodemographic variables we selected—such as migration background, experiences of discrimination, and German language skills—are likely interrelated, k-means was better suited for our analysis. K-means clusters participants based on overall similarity, does not require the assumption of conditional independence, and allows for more flexible and scalable grouping of diverse and potentially correlated variables [52]. Categorical variables are integrated into the analysis via dummy coding (one-hot encoding), converting categorical variables into binary vectors. Responses such as ‘not stated’ or ‘don’t know’ were treated as a separate category in the cluster analyses to preserve these participants’ data and because they contain meaningful information. True missing values (NA) were excluded. K-means is an efficient and scalable clustering method, ideal for handling the data types present in our dataset. To determine the optimal number of clusters, we referred to the Scree plot (Elbow Criterion). For graphical aid, we refer to silhouette values [53]. Silhouette values below 0.3 indicate a poor fit, between 0.3 and 0.5 a fair fit, and values above a good fit [54]. We further evaluated the stability of the clustering solution using gap statistics [55] and cluster stability analyses, with clusterwise Jaccard bootstrap analysis [55–57]. We performed Principal Component Analysis (PCA) to detect outliers, as k-means can be sensitive to these. We applied z-scores with a threshold of *SD* = 3 for detection.

To analyze the associations between socio-demographic and other variables with ED risk, we used the following variables: [1] socio-demographic clusters [2], S-HS [3], S-SES, and both scales of body dissatisfaction [4] BD-F and [5] BD-M. Participants with true missing values (NAs) were excluded from the regression analyses. We began with a full model that included all variables and then used a backward stepwise selection process to identify the best-fitting model. We selected the final model based on the Akaike Information Criterion (AIC) and Bayesian Information Criterion (BIC) values. Reported results are from the optimized model. To account for multicollinearity we calculated the Variance Inflation Factor (VIF); VIF > 5 indicates severe mulicollinearity [58]. In the Appendix, we have added a multiple regression analysis, in which each of the 10 variables is considered as an isolated variable. This serves to compare the results with those of the cluster analysis. However, we note that these isolated analyses are limited by the relatively small variance for some categories.

## Results

### Sample characteristics

A total of 304 individuals participated in the survey. Six participants stated that they had already participated once and were thus excluded, resulting in a sample of *N* = 298. Regarding gender, 290 out of 298 participants identified as women. The other 8 participants identified as women, as required for study participation, but also indicated a diverse gender identity (*n* = 6) or did not specify (*n* = 2) in the corresponding question on gender identity. Our sample of 298 participants had a mean age of 28.4 years (*SD* = 9.45; *range* = 18–64), and most participants (64.8%) reported being predominantly attracted to men. Most participants had German as their first language (82.9%). The majority reported having no migration history (69.8%), and 86.2% identified as not being part of a minority group. Regarding education, the majority (84.2%) had attended school for at least 12 years. Most participants were single (77.6%). Additionally, 85.6% reported not participating in informal care work, and 95% indicated they did not have a disability. The exact numbers (*N*,* %*,* mean*,* SD*) for all categories are displayed in the Appendix in Table A1.

### Cluster analysis

We began our cluster analysis with data from the 298 individuals. The Elbow criterion led to an optimal number of clusters of *k* = 3. With PCA, we identified seven outliers and repeated our analysis with the remaining *N* = 291 participants. The average silhouette score for this final solution was 0.46, which is a fair fit. Also, the gap statistic indicated that k = 3 (gap = 0.708, SE = 0.008) is a plausible solution. Mean Jaccard indices were high across clusters (Cluster 1: 0.915; Cluster 2: 0.946; Cluster 3: 0.838). In the Appendix, Figure A1 presents the composition of each of the three clusters based on socio-demographic variables. Figure A2 shows the results of the Elbow method. Figure A3 provides a PCA plot illustrating the cluster assignments and marked outliers. Figure A4 presents the silhouette plot for the final clustering solution.

We present the clusters in order of increasing ED risk of the EAT-8: The cluster with the lowest risk, ***Cluster One*** (*n* = 42 individuals), consisted of 100% of individuals over 30 years of age. 11.6% reported a disability, and 45.2% reported informal caregiving responsibilities. Half of the individuals were married/in a partnership, while 14.3% reported being divorced and 35.7% reported being single. Regarding sexual orientation, 73.8% defined themselves as heterosexual. Less than one-fifth reported a migration history (19.1%), and only a low proportion identified as part of an ethnic minority or a racialized group (2.4%). ***Cluster Two*** (*n* = 201 individuals) was primarily composed of younger (19–29 years of age) individuals (85.1%) without disabilities (97%), without informal caregiving responsibilities (94%), and mostly single (95%). 20% reported a migration history, and only 7% identified as part of an ethnic minority or a racialized group. This cluster included the 1% (2 individuals) who identified as having a diverse gender identity. While 66.2% identified as heterosexual, 4.5% reported attraction to women, and 25.4% to both men and women, representing the highest proportion of bisexual individuals among all clusters. ***Cluster Three*** (*n* = 48 individuals) was split into 54.2% younger individuals (18–19 years) and 45.8% older individuals (30–50 + years). While 77.1% of individuals in this cluster did not have informal caregiving responsibilities and 95.8% did not report any disabilities, the cluster was characterized by non-native German speakers (100%). As this cluster had a large proportion identifying themselves as having a migration history (83.3%), this group had a high proportion of respondents who identified themselves as being part of a minority (20%) or were not sure about this (20%). Only 60% identified as heterosexual, indicating a more diverse sexual orientation profile. The exact numbers (*n*,* %*,* mean*,* SD*) for all socio-demografics for each cluster are displayed in the Appendix in Table A2.

### ED Risk using the EAT-8 and the EDE-Q

One participant was excluded due to missing data, resulting in a final sample of *N* = 290 for regressions. The best-fitting regression models both eliminated S-SES as a predictor of ED risk, while all other variables remained (Cluster assignment, BD-F, BD-M and S-HS). Table 1 presents the results of the person-centered cluster-based multiple regression analysis, including the odds ratios (OR) with CI for each predictor for the outcome EAT-8. Table 2 presents the same analysis for the outcome of the EDE-Q. VIF indicated no concern regarding multicollinearity among the variables. Cluster assignment, BD-F, and BD-M are associated with the risk of developing an ED. The results differ in that Cluster Two showed significantly higher ED risk on the EAT-8, but not on the EDE-Q. On the other hand, S-HS was significantly associated with the EDE-Q, but not with the EAT-8. Similarly was that Cluster Three consistently exhibited significantly increased risk across both measures, with odds ratios of 4.38 for the EAT-8 and 3.0 for the EDE-Q compared to Cluster One. These associations were independent of BD-F and BD-M.

**Table 1:** Results of the Multiple Logistic Regression Model for the EAT-8

	Estimate	Std. Error	*P* value	Odds Ratio CI (95%)
Cluster One (Intercept)	− 0.18	0.94	0.8505	0.84 CI = [0.13; 5.38]
Cluster Two	1.03	0.43	0.0183 *	2.80 CI = [1.19; 6.68]
Cluster Three	1.48	0.58	0.0106 *	4.38 CI = [1.45; 14.13]
BD-F	1.34	0.24	3.52e-08 ***	3.82 CI = [2.43; 6.31
BD-M	0.46	0.23	0.0416 *	1.58 CI = [1.03; 2.51]
S-HS	− 0.38	0.21	0.0672	0.68 CI = [0.45; 1.02]

EAT-8 at risk ≥ 2. Bold values indicate statistical significance: *** p < 0.001, ** p < 0.01, * p < 0.05; Akaike Information Criterion (AIC): 287.71; Bayesian Information Criterion (BIC): 309.73

**Table 2:** Results of the Multiple Logistic Regression Model for the EDE-Q

	Estimate	Std. Error	*P* value	Odds Ratio CI (95%)
Cluster One (Intercept)	− 1.05	1.01	0.30	0.35 CI = [0.04; 2.50]
Cluster Two	0.93	0.48	0.054 .	2.54 CI = [1.102; 6.84]
Cluster Three	1.47	0.58	0.011 *	3.00 CI = [1.42; 13.78]
BD-F	1.58	0.24	4.25e-11 ***	4.95 CI = [3.11; 7.87]
BD-M	0.46	0.21	0.026 *	1.60 CI = [1.06; 2.44]
S-HS	− 0.78	0.23	0.000593 ***	0.46 CI = [0.29; 0.71]

*EDE-Q at risk* ≥ *2.4*. Bold values indicate statistical significance: *** p < 0.001, ** p < 0.01, * p < 0.05; Akaike Information Criterion (AIC): 262.22; Bayesian Information Criterion (BIC): 284.22

**Fig. 1:**
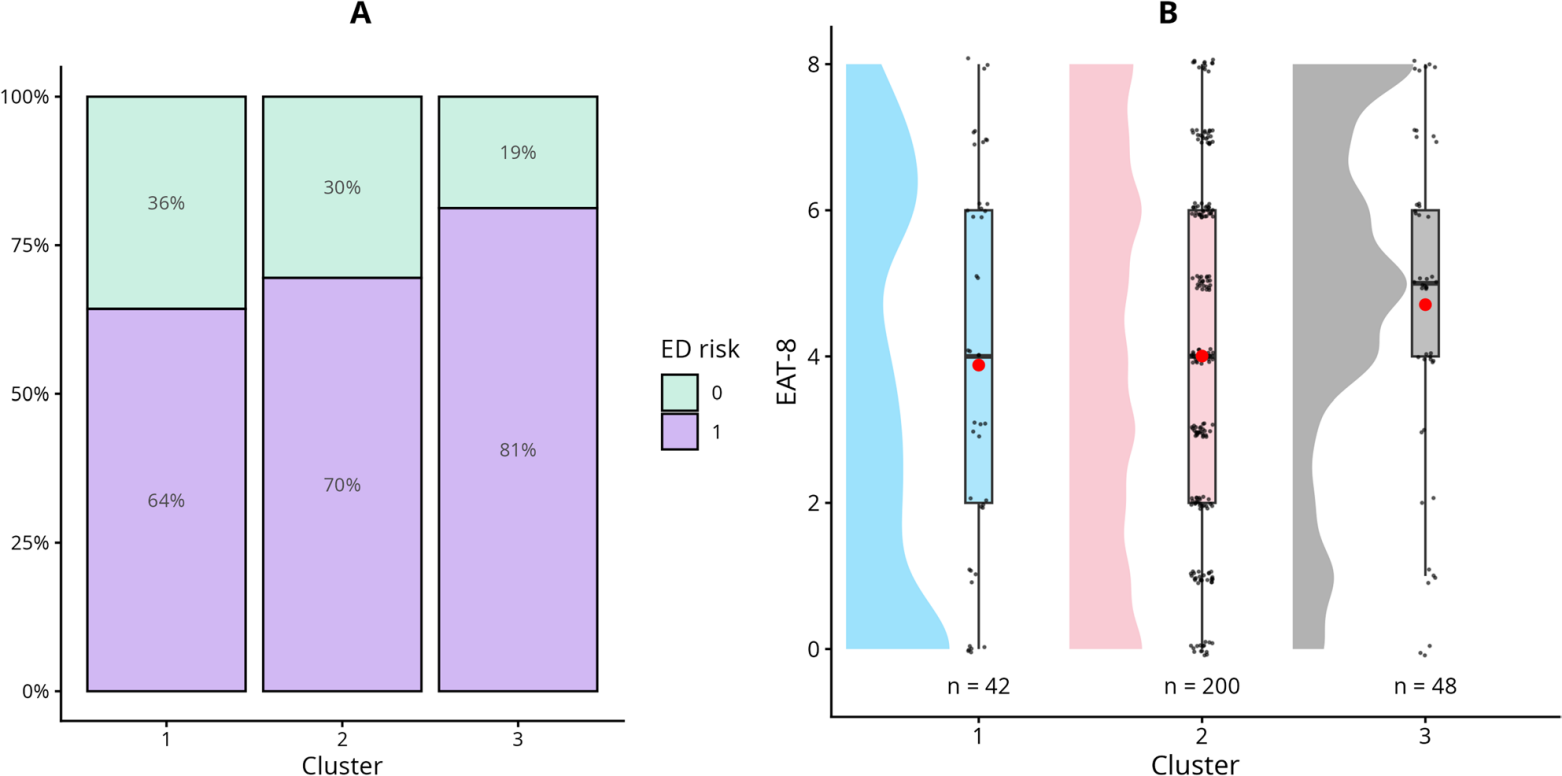
Visualisation of the distribution of ED risk and EAT-8 scores across clusters. **A** Percentage distribution of ED risk based on the EAT-8 across the three clusters. **B** Distribution of EAT-8 scores within each cluster. Red markers indicate cluster means. In women, a score of ≥ 2 indicates elevated risk for an ED

**Fig. 2:**
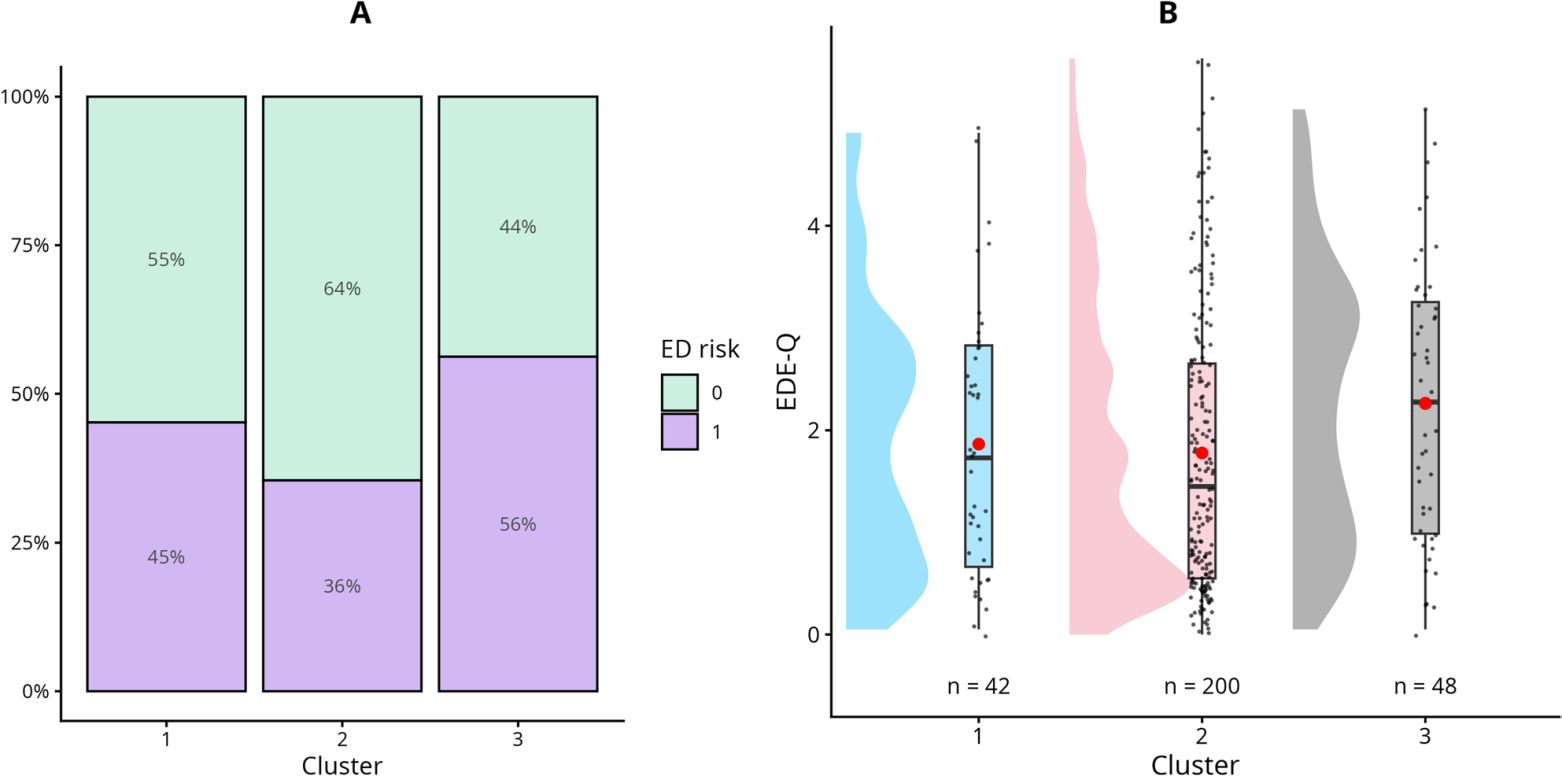
Visualisation of the distribution of ED risk and EDE-Q scores across clusters. **A** Percentage distribution of ED risk based on the EDE-Q across the three clusters. **B** Distribution of EDE-Q scores within each cluster. Red markers indicate cluster means. In women, a score of ≥ 2.4 indicates elevated clinical risk for an ED

The analysis aimed to examine whether person-centered socio-demographic clusters could serve as meaningful risk indicators for EDs, rather than isolated socio-demographic factors. Although our sample was relatively homogeneous, with limited variability in the 10 socio-demographic variables, we still observed notable differences in ED risk across clusters. Figures 1 and 2 illustrate the distribution of ED risk across the three socio-demographic clusters. As expected, the results show that the proportion of participants classified as “at risk” was higher for the EAT-8 than for the EDE-Q. Looking at the EDE-Q distribution of the clusters, the distribution of Cluster Three appears to be shifted upwards compared to Cluster One. Looking at EDE-Q in contrast to EAT-8, differences can be seen in Cluster Two, which could illustrate the focus of the two measurements.

**Fig. 3:**
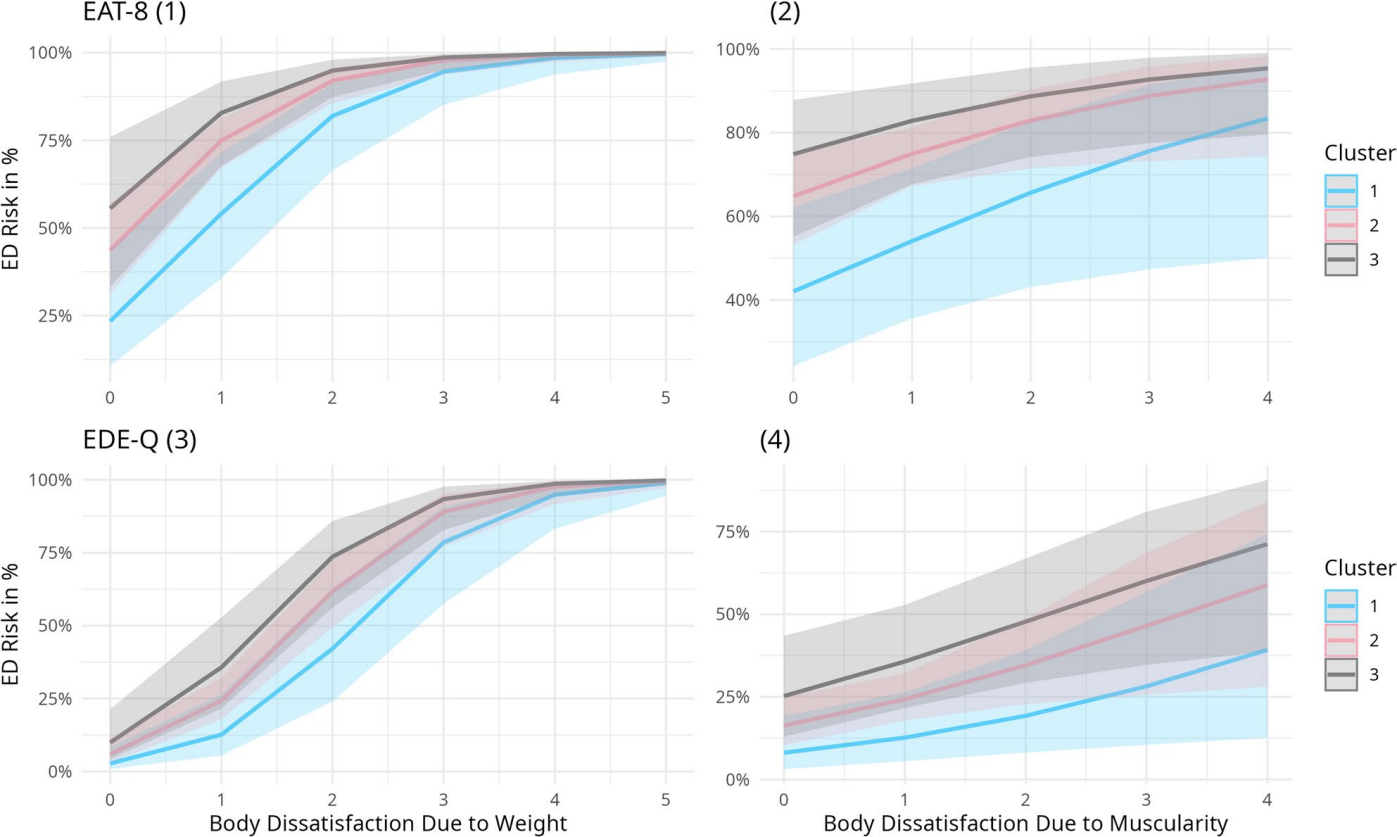
Associations between ED risk and body dissatisfaction across clusters. Panels show the relationship between ED risk and body dissatisfaction for (1) body fat and (2) muscularity based on the EAT-8, and (3) body fat and (4) muscularity based on the EDE-Q across the three clusters. Values of 0 indicate no discrepancy, while higher values (up to 5) reflect greater body dissatisfaction in either direction (i.e., higher or lower body fat or muscularity)

To visualize the effects of Cluster and body dissatisfaction, Fig. 3 further examines the relationship between ED risk and both forms of body dissatisfaction in all Three Clusters. Regarding BD-F, the association with ED risk was particularly strong in women with high body dissatisfaction. BD-F in the EAT-8, as well as the EDE-Q, was 100% in all three clusters (see Fig. 3(1;3)). BD-F in association with EDE-Q follows an S-Curve in all three Clusters (see Fig. 3(3)). BD-M appeared as a novel driver of ED risk, even if the association with muscularity concerns was not as strong as for body fat, with a gradual increase across clusters (see Fig. 3(2;4)).

Given the moderate correlation (*r* = 0.46) between BD-F and BD-M, we re-ran our regression analysis excluding BD-M to ensure the robustness of the results and assess the relevance of the socio-demographic clusters. The estimates, which can be found in the Appendix (Table A3), indicate that the socio-demographic clusters remain significantly associated with the outcome, even after excluding BD-M. Additionally, we reintroduced S-SES to confirm its lack of predictive power for ED risk within our sample (Table A4). Lastly, we conducted a multiple regression analysis using the socio-demographic variables in isolation. In this analysis, no variable was significantly associated with ED risk (Table A5).

## Discussion

Our study examined how socio-demographic clustering, body dissatisfaction, and subjective health are associated with ED risk in women, using two established screening instruments (EAT-8 and EDE-Q). The findings must be interpreted within the constraints of our sample, which was predominantly young, highly educated, heterosexual, without a migration history, and recruited through an online survey and university mailing list. Importantly, clustering goes beyond additive models of cumulative risk. In this sample, the cluster-based approach identified three socio-demographic patterns associated with different levels of risk for ED. The identified clusters should not be interpreted as direct representations of structural oppression; rather, they reflect how structural and social stressors may be expressed as lived patterns of psychosocial vulnerability.

Across both ED risk measurements, Cluster Three consistently had the highest proportion of individuals with a risk of developing an ED. This cluster was characterized by a higher proportion of participants with a migration history, non-native German language use, minority group identification, lower educational attainment, and a more diverse pattern of sexual orientation. In the context of EDs, being part of a minority group may reflect increased psychosocial vulnerability, such as exposure to minority-specific body ideals, subclinical disordered eating patterns, or stress related to identity navigation, rather than direct effects of structural discrimination [18] providing a plausible mechanism for our interpretation of Cluster Three as a psychosocially vulnerable group. Examining marginalized populations is particularly important because, despite often reporting comparable or higher symptom levels, these groups are less likely to receive appropriate and timely treatment [59] and discrimination (e.g., based on language barriers) and internalized stigma might contribute to even more elevated rates of disordered eating.

In contrast, Cluster One showed significantly lower ED risk than Cluster Three across both ED risk measures. Cluster One was predominantly composed of older individuals, with a higher proportion of participants reporting disability and informal caregiving responsibilities. It could be suggesting that not all forms of social burden uniformly increase ED risk; some constellations of disadvantage may instead be more strongly associated with other mental health outcomes. Indeed, previous research shows that becoming a caregiver can negatively impact general health and well-being [60] and is more associated with specific forms of ED risk, e.g., emotional eating and not EDs in general [61]. Nevertheless, a substantial proportion of participants in this cluster were classified as at risk: 67% according to the EAT-8 and 33% according to the EDE-Q.

Cluster Two displayed a more nuanced profile across the two ED measures. While the EAT-8 indicated elevated risk compared to Cluster One, this was not reflected in the EDE-Q scores. This discrepancy may point to differences in the constructs assessed by each instrument, with the EAT-8 capturing broader eating attitudes and restrictive or dieting-related behaviors, and the EDE-Q focusing more specifically on core ED pathology, including binge eating, compensatory behaviors, and weight and shape concerns. The elevated EAT-8 scores in Cluster Two may therefore reflect emerging or subclinical patterns of disordered eating that have not yet developed into the more severe symptomatology assessed by the EDE-Q. In this sense, the EAT-8 scores may represent early warning signs or a specific vulnerability. Within this realm, previous research has shown that body dissatisfaction and restrictive eating behaviors may precede the onset of EDs [62]. Also, early compensatory strategies and cognitive symptoms often emerge before diagnosable EDs develop, and targeted prevention programs have been shown to be effective at this stage [63]. Therefore, we may assume that the differing results may mirror differences in vulnerability, attitudes, and early risk processes, rather than ED severity levels. Cluster Two may thus represent a group for which preventive interventions may be especially relevant. Additionally, this cluster was composed primarily of younger, mostly single participants with no migration history. Recent research suggests that loneliness can influence disordered eating, but relationship status alone does not moderate this link [64], pointing to another avenue for intervention [64].

Both fat-related (BD-F) and muscularity-related (BD-M) body dissatisfaction were independently associated with higher ED risk, indicating that contemporary body ideals among women extend beyond thinness. The stronger association between fat-related dissatisfaction is consistent with traditional ED research, while the additional role of muscularity-related dissatisfaction aligns with emerging evidence that fitness- and strength-oriented body ideals are increasingly relevant for women. These findings support calls to incorporate more diverse body image dimensions into ED risk models and diagnostics. The odds ratio for BD-M in the EAT-8 regression and in the EDE-Q, at 1.6, confirms the relevance of muscularity concerns in the clinical setting.

Taken together, the findings suggest that ED risk in women is shaped by both social positioning, here captured through socio-demographic variables, and body-related concerns. Importantly, these associations should not be construed as causal. Instead, they highlight constellations that can inform future longitudinal research. Multiple studies have argued that health research should not treat social categories independently, but rather consider how intersecting social identities and social positions can jointly shape health outcomes [25, 52]. Clinicians should consider intersectional socio-demographic factors when screening for ED risk.

### Further research

Subsequent studies should examine these clusters in more diverse and representative populations and directly test potential mediating mechanisms, such as discrimination experiences, social exclusion, identity-related stress, migration-related stress, and barriers to healthcare access. Greater attention to intersectional constellations—such as queer women with migration histories or sexual minority individuals with lower educational attainment or lower subjective social status—may clarify how layered social positions interact to shape ED risk and support the development of culturally sensitive, structurally informed prevention and intervention strategies. Since we obtained different results for the EAT-8 and the EDE-Q in Cluster Two, our findings underscore the importance of a closer examination of subclinical manifestations of EDs, e.g., if embodiment disturbances represent an intermediate factor that helps explain group differences. A previous study has shown that embodiment disturbances were most pronounced in patients with anorexia nervosa, while individuals with gender dysphoria also exhibited elevated levels compared to the general population [65]. Future research should investigate how socio-demographic and embodiment variables interact with early attitudes and eating behaviors. Furthermore, since our results showed different risk profiles depending on the assessment instrument, our findings may suggest that the choice -or combination- of assessment instruments is crucial to accurately capture different stages and levels of ED risk.

While we formed clusters within our sample of women to analyze the association with and contrary to the view that EDs are primarily a woman´s issue, there is evidence that EDs occur in men more frequently than assumed [66] only seeking less help, highlighting this as an important area for future research [5]. Concerning gender, a larger-scale study would be desirable to distinguish between transgender and cisgender individuals. For example, transgender individuals report significantly higher rates of ED symptoms compared to their cisgender peers [67]. The authors suggest that these individuals are motivated to lose or gain weight to achieve a more gender-affirming body shape. The intersections between gender dysphoria-defined as psychological distress resulting from a mismatch between gender identity and assigned sex at birth-body image concerns, and EDs require greater attention [68].

Greater attention should be directed to specific forms of body dissatisfaction, as well as to the social positions and processes in which they emerge. For example, dissatisfaction related to muscularity may be more strongly associated with certain social roles or gendered expectations, whereas dissatisfaction with weight may be linked to different social positions and experiences, including exposure to weight-related stigma. Differences can be explained by underlying mechanisms such as social comparison processes, which may occur more frequently in an online context and thus further exacerbate concerns about one’s own body image [69]. It is therefore important to examine how social media differentially promotes ideals of muscularity versus thinness, highlighting the need to further differentiate between fat-related and muscularity-related dissatisfaction in future research.

### Limitations

We conducted an extended socio-demographic assessment to enhance diversity measurement in research. However, we did not include all dimensions suggested by the DIMIS [13]. We excluded the variable of “religion”/“worldview” due to space constraints and survey length. Additionally, we assessed gender using the question ‘Which gender do you identify with?’ (female, male, diverse). While male participants were excluded from the study, those identifying as ‘diverse’ were grouped into a single category, which does not capture the unique experiences of non-binary and gender non-conforming individuals. Future studies should address this by assessing specific gender identities separately. Recommendations can be found in [70].

Additionally, our focus was limited to ED risk. While the EAT-8 [45] and the EDE-Q are important screening tools, they do not replace a clinical diagnosis, leaving it unclear whether socio-demographics play a similar role among women with clinically diagnosed EDs. Moreover, existing assessment tools for EDs in men warrant improvement, as symptom presentation may differ [27].

We identified three clusters that showed fair separation [53] but unequal cluster sizes. There is a risk that Clusters One (*n* = 42) and Three (*n* = 48) could act as residual categories, grouping participants who deviate from Cluster Two, the “dominant” cluster (*n* = 201), without forming a clearly „separate“ pattern. Small clusters also present statistical challenges: estimates of means and variances are less stable, outliers can have a disproportionately large influence, and reproducibility in independent samples may be limited. Additionally, the relatively small sample sizes of Clusters One and Three in relation to the number of included variables (*n* = 10) increase the risk of instability and potential overfitting. Although our analyses showed acceptable stability and outliers were examined using PCA, statistical uncertainties due to unequal cluster sizes cannot be ruled out. Statistically speaking, caution is advised when generalizing the results. Likewise, we do not want the results to be interpreted as definitive sociological types, but rather as an exploratory, data-driven profile that describes patterns within this specific sample. Smaller, but internally consistent clusters can reveal meaningful correlations with relevant characteristics. Future studies should therefore aim for larger and more balanced groups to stabilize our results.

Additionally, over half of the participants were classified as at-risk, which may have biased the results. Our sample may have been biased, as participants with a strong personal interest in the topic might have been more likely to participate. It is also possible that the EAT-8 is oversensitive in categorizing individuals as at risk for ED [71] Participants completed the study online and remotely, which may have introduced selection effects and reduced data accuracy. Online survey platforms are generally associated with higher proportions of low-quality responses [24], particularly among individuals who use study participation as their primary source of income [72]. Online survey platforms may be susceptible to automated or bot responses. Although Prolific implements actions to minimize this risk, and we did include an attention check, we cannot completely rule out the possibility that bots were used. The graphics used (FBS and FFITBS scales) may reflect stereotypical body ideals among women and may not fully capture diverse experiences across the gender spectrum. Additionally, the images are Caucasian, which may limit the applicability of the scales in studies focusing on intersectional or ethnically diverse populations [73]. Finally, given the cross-sectional nature of the study, causal interpretations should be made cautiously.

## Conclusion

Cluster-based analyses revealed distinct psychosocial vulnerability profiles, underscoring the need to move beyond “one-size-fits-all” approaches to evaluations of individuals “at-risk“ for EDs. The independent roles of both fat-related and muscularity-related body dissatisfaction highlight that contemporary ED risk in women extends beyond thinness alone. These findings support more nuanced, intersectional, and body-inclusive approaches to ED research and intervention.

## Supplementary Information

Below is the link to the electronic supplementary material.

Supplementary Material 1.

## Data Availability

The data and code that support the findings of this study are available from the corresponding author upon request.

## Abbreviations

AIC: Akaike Information Criterion
BD-F: Body dissatisfaction regarding body fat
BD-M: Body dissatisfaction regarding muscularity
BIC: Bayesian Information Criterion
BMI: Body Mass Index
CI: Confidence interval
DiMIS: Diversity Minimal Item Set
ED: Eating disorder
FBS: Female Body Scale
FFITBS: Female Fit Body Scale
OR: Odds ratio
OSF: Open Science Framework
PCA: Principal Component Analysis
SES: Socioeconomic status
‘SWAG’: ‘skinny, white, affluent, girls’
VIF: Variance Inflation Factor
