# Supplementary material for: Associations between socio-demographic patterns, body dissatisfaction, and eating disorder risk in women: a cluster-based approach
Source: J Eat Disord. 2026 May 11;14:112. doi: 10.1186/s40337-026-01616-8 (PMC13173984; doi:10.1186/s40337-026-01616-8)
Supplement: Supplementary file 1 — Supplementary Material 1. [file 40337_2026_1616_MOESM1_ESM.docx]

**Appendix**


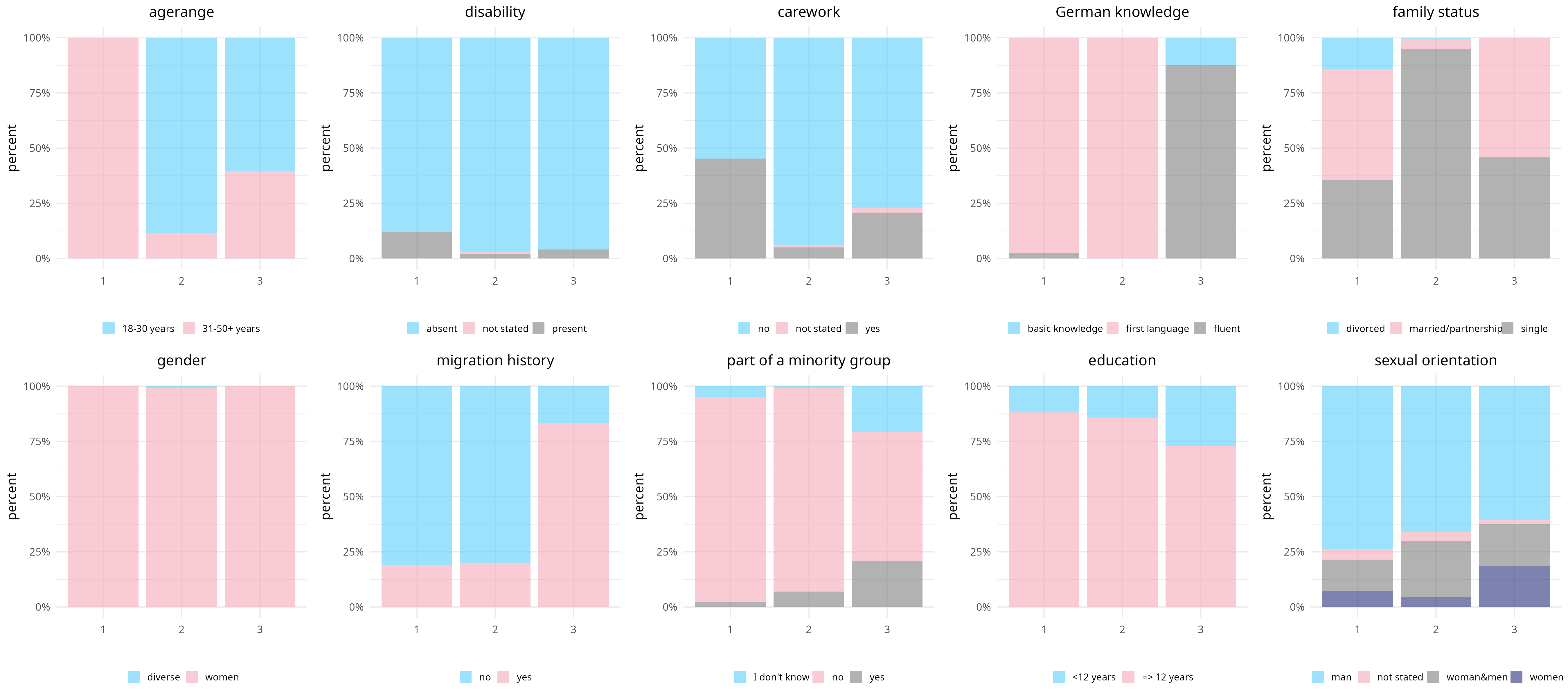


**Figure A1.** **Proportional Representation of Clusters based on the Ten Socio-Demographic Variables*.*** Only for clarity in visual presentation, we categorized age as a binary variable, following an approach defining individuals aged 18–29 as the younger age group (74).

**Table A1** Socio-demographic characteristics of participants (N = 298**)**

| **Variable** | N (%) | **Variable** | N (%) |
| --- | --- | --- | --- |
| Identified gender |  | Part of a minority group |  |
| Women | 290 (97.37) | No | 261 (85.85) |
| Diverse | 6 (1.97) | Yes | 27 (8.88) |
| Not specified | 2 (0.66) | I don’t know | 16 (5.26) |
| Mean age (SD, Range) | 28.40 (9.45, 18 - 64) | Education (years of school) |  |
| Attracted to (sexual orientation): |  | At least 12 years | 256 (84.21) |
| Men | 197 (64.80) | Less than 12 years | 48 (15.79) |
| Women | 24 (7.89) | Marital status /partnership |  |
| Men and women | 70 (23.03) | Single | 236 (77.63) |
| Other genders | 2 (0.66) | Married/with partner | 59 (19.41) |
| Not specified | 11 (3.62) | Divorced | 9 (2.96) |
| German  knowledge |  | Informal care work |  |
| First language | 253 (83.22) | Yes | 41 (13.49) |
| Fluent | 45 (14.8) | No | 260 (85.53) |
| Basic knowledge | 6 (1.97) | Not stated | 3 (0.99) |
| Migration history |  | Disability |  |
| No | 213 (69.8) | Yes | 13 (4.28) |
| Yes | 91 (30.2) | No | 289 (95.07) |
|  |  | Not stated | 2 (0.66) |

Values are reported as n (%) or mean


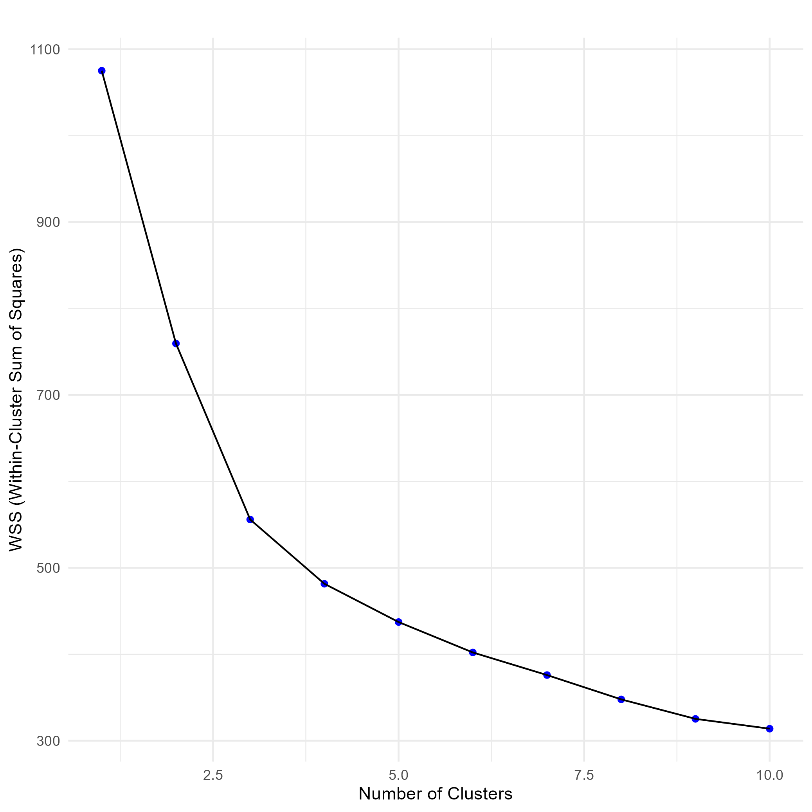


**Figure A2.** **Elbow Method for Determining Optimal Number of Cluster.** The Elbow Method is a technique used to determine the optimal number of clusters by plotting the Within-Cluster Sum of Squares (WSS) against the number of clusters.


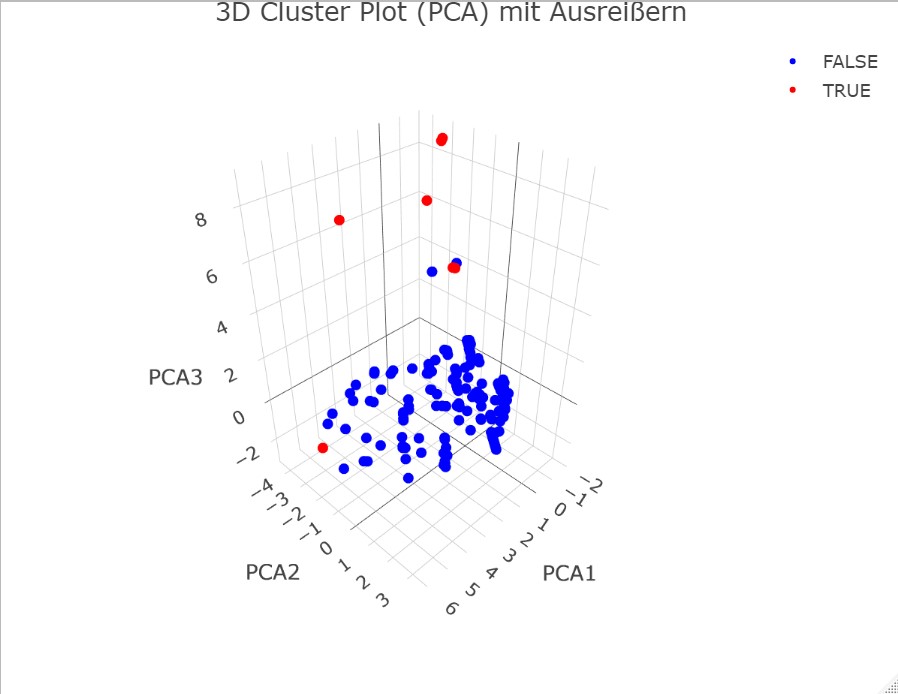

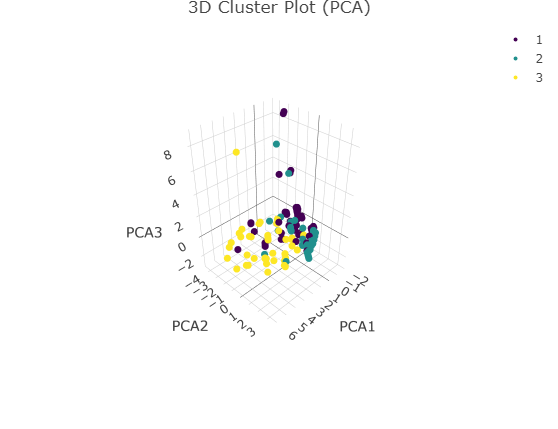


*(a) (b)*

**Figure A3. Principal Component Analysis (PCA) Plot with (a) Cluster Assignment and (b) with Marked Outliers.** (a) Cluster One is shown in purple, Cluster Two in green, and Cluster Three in yellow. (b) Outliers are marked in red.


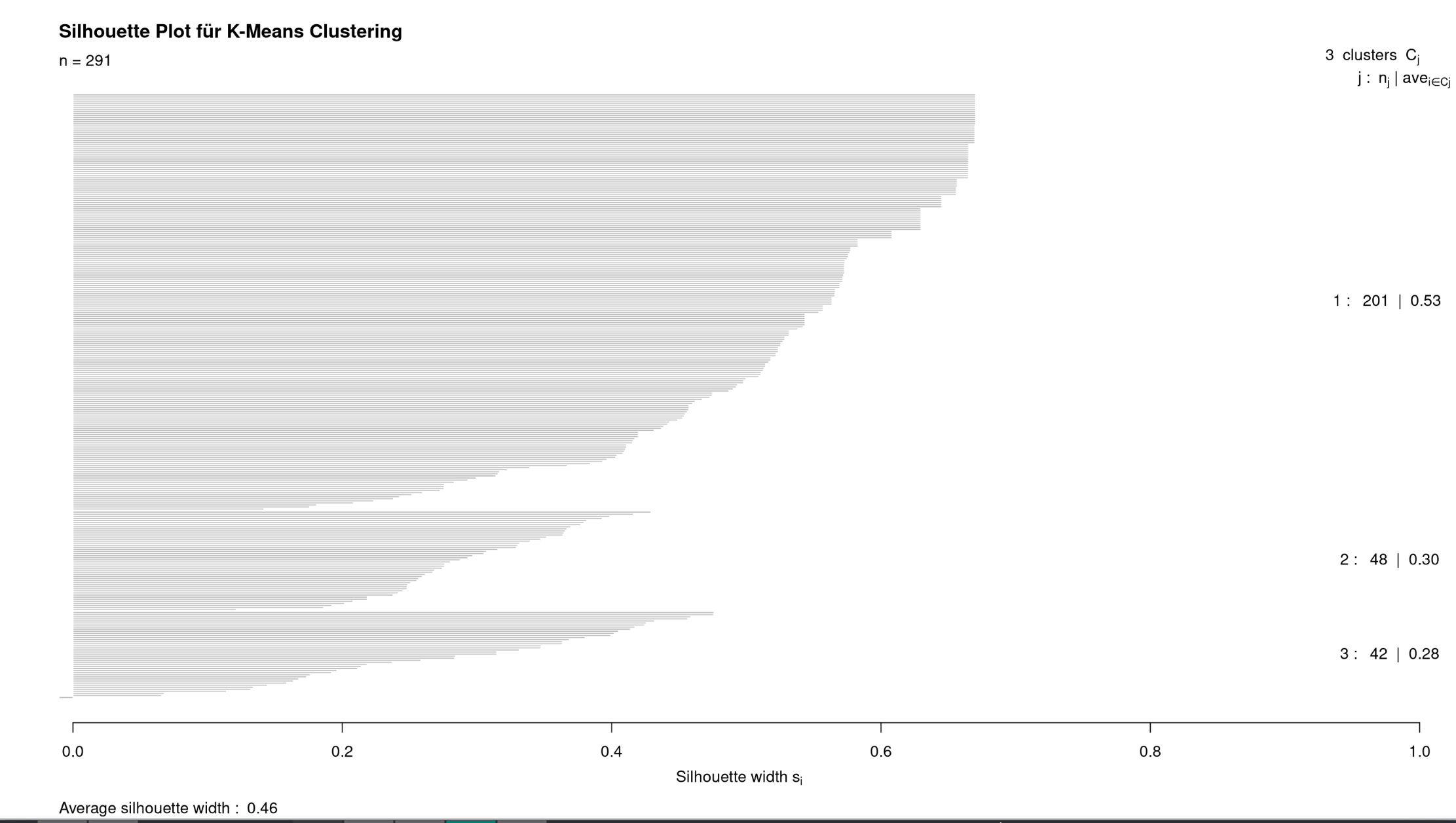


**Figure A4. Silhouette Plot for the Three-Cluster Solution.**

**Table A2** Socio-demographic characteristics of participants (N = 291**)** by Cluster

| **Variable** | **Cluster One** | **Cluster Two** | **Cluster Three** |  | **Variable** | **Cluster One** | **Cluster Two** | **Cluster Three** |
| --- | --- | --- | --- | --- | --- | --- | --- | --- |
|  | *n* = 42 | *n* = 201 | *n* = 48 |  |  | *n* = 42 | *n* = 201 | *n* = 48 |
| Mean age (SD) | 45.83 (9.22) | 24.29 (4.18) | 30.12 (7.71) |  | Part of a minority group |  |  |  |
| Identified gender |  |  |  |  | Yes | 1 (2.4%) | 14 (7.0%) | 10 (20.4%) |
| Women | 42 (100%) | 199 (99.0%) | 48 (100%) |  | I don’t know | 2 (4.8%) | 2 (1.0%) | 10 (20.4%) |
| Diverse | 0 (0%) | 2 (1.0%) | 0 (0%) |  | Education (years of school) |  |  |  |
| Attracted to (sexual orientation): |  |  |  |  | At least 12 years | 37 (88.1%) | 172 (85.6%) | 35 (72.9.%) |
| Men | 31 (73.8%) | 133 (66.2%) | 29 (60.4%) |  | Less than 12 years | 5 (11.9%) | 29 (14.4%) | 13 (27.1%) |
| Women | 3 (7.1%) | 9 (4.5%) | 9 (18.8%) |  | Marital status /partnership |  |  |  |
| Men and women | 6 (14.3%) | 51 (25.4%) | 9 (18.8%) |  | Single | 15 (35.7%) | 191 (95.0%) | 22 (45.8%) |
| Not specified | 2 (4.8%) | 8 (4.5%) | 1 (2.1%) |  | Married/with partner | 21 (50.0%) | 9 (4.5%) | 26 (54.2%) |
| German  knowledge |  |  |  |  | Divorced | 6 (14.3%) | 1 (0.5%) | 0 (0%) |
| First language | 41 (97.6%) | 201 (100%) | 0 (0%) |  | Informal Care Work |  |  |  |
| Fluent | 1 (2.4%) | 0 (0%) | 42 (87.5%) |  | No | 23 (54.8%) | 189 (94.0%) | 37 (77.1%) |
| Basic knowledge | 0 (0%) | 0 (0%) | 6 (12.5%) |  | Yes | 19 (45.2%) | 10 (5.0%) | 10 (20.8%) |
| Migration history |  |  |  |  | Not Stated | 0 (0%) | 2 (0.5%) | 1 (2.1%) |
| No | 34 (81.0%) | 161 (80.1%) | 8 (16.7%) |  | Disability |  |  |  |
| Yes | 8 (19.0%) | 40 (19.9%) | 40 (83.3%) |  | Yes | 5 (11.6%) | 4 (2.0%) | 2 (4.2%) |
| Part of a minority group |  |  |  |  | No | 37 (88.1%) | 195 (97.0%) | 46 (95.8%) |
| No | 39 (92.9%) | 185 (92.0%) | 28 (58.3%) |  | Not Stated | 0 (0%) | 2 (1.0%) | 0 (0%) |

Values are reported as n (%) or mean**Table A3** Results of the Multiple Logistic Regression Model without BD-M

|  | *Estimate* | *Std. Error* | *P value* |
| --- | --- | --- | --- |
| Cluster One (Intercept) | 0.12 | 0.92 | 0.89765 |
| Cluster Two | 1.04 | 0.43 | 0.01603 * |
| Cluster Three | 1.49 | 0.57 | 0.00819 ** |
| BD-F | 1.49 | 0.24 | 2.17e-10 *** |
| S-HS | -0.41 | 0.21 | 0.04996 * |

Signif. codes: 0 ‘***’ 0.001 ‘**’ 0.01 ‘*’ 0.05 ‘.’ 0.1 ‘ ’ 1; AIC: 28

**Table A4** Results of the Multiple Logistic Regression Model with S-SES

|  | *Estimate* | *Std. Error* | *P value* |
| --- | --- | --- | --- |
| Cluster One (Intercept) | 0.23 | 1.03 | 0.8243 |
| Cluster Two | 1.03 | 0.43 | 0.0194 * |
| Cluster Three | 1.49 | 0.58 | 0.0106 * |
| BD-F | 1.32 | 0.24 | 6.99e-08 *** |
| BD-M | 0.45 | 0.22 | 0.0477 * |
| S-SES | -0.11 | 0.11 | 0.3329 |
| S-HS | -0.31 | 0.22 | 0.1655 |

Signif. codes: 0 ‘***’ 0.001 ‘**’ 0.01 ‘*’ 0.05 ‘.’ 0.1 ‘ ’ 1; AIC: 288.77

**Table A5** Results of the Multiple Logistic Regression Model With Isolated Socio-demographic Factors

|  | *Estimate* | *Std. Error* | *P value* |
| --- | --- | --- | --- |
| Intercept | 1.92 | 1.43 | 0.18365003 |
| Age | 0.01 | 0.02 | 0.75619873 |
| Sexual orientation (attracted to men and women) | -1.20 | 0.73 | 0.07995541 |
| Sexual orientation (attracted to men) | -1.22 | 0.69 | 0.05207332 |
| Education (less than 12 years of schooling) | 0.49 | 0.40 | 0.20629001 |
|  |  |  |  |
| German (first language) | 0.46 | 0.52 | 0.38719623 |
| Migration history (No) | -0.73 | 0.43 | 0.08764240 |
| Part of a minority group (No) | 0.01 | 0.58 | 0.98851094 |
|  |  |  |  |
| Disability (No) | 0.54 | 0.68 | 0.43760001 |
|  |  |  |  |
| Informal care work (No) | -0.59 | 0.48 | 0.21411324 |
| Marital Status (married/with partner) | -0.05 | 0.48 | 0.92525416 |

Categorical variables were cleaned prior to modeling by combining small categories and removing missing. “German basic knowledge” was merged into the “fluent” category, and “divorced” marital status was merged into “single.” Due to a low number of events, Firth’s penalized likelihood correction (via the logistf R package) was applied to reduce bias. Model fit and coefficient significance were assessed using Wald and likelihood-ratio tests. LRT = 11.21 on 10 df, p = 0.341; Wald test = 44.92 on 10 df, p = 2.25e-06; n = 263
